# Use of Intranasal Analgesia for Acute Pain in Pediatric Emergency Departments: A Systematic Review and Meta‐Analysis of Randomized Controlled Trials

**DOI:** 10.1155/prm/1894053

**Published:** 2026-10-19

**Authors:** Mohammed Alsabri, Yahya A. Mahmoud, Shree Rath, Eslam Abady, Mohamed Ashraf Shehab, Raseel Sleem, Luis L. Gamboa, Mohammed Hamzah, Michael Gillman

**Affiliations:** ^1^ Emergency Department Althawra Modern General Hospital Sana’a Yemen; ^2^ Pediatric Emergency Department Department of Pediatrics St. Christopher’s Hospital for Children Philadelphia Pennsylvania USA stchristophershospital.com; ^3^ Faculty of Medicine Al-Azhar University Gaza State of Palestine azhar.edu.eg; ^4^ All India Institute of Medical Sciences (AIIMS) Bhubaneswar India aiims.edu; ^5^ Faculty of Medicine Tanta University Tanta Egypt tanta.edu.eg; ^6^ Faculty of Medicine Menoufia University Menoufia Egypt menofia.edu.eg; ^7^ Assistant Professor of Pediatrics Drexel University College of Medicine Philadelphia Pennsylvania USA drexel.edu; ^8^ Program Director Pediatric Emergency Medicine Fellowship St. Christopher’s Hospital for Children Philadelphia Pennsylvania USA stchristophershospital.com; ^9^ Department of Pediatric Cardiology Advocate Children’s Hospital Oak Lawn Illinois USA advocatechildrenshospital.com; ^10^ Department of Pediatric Emergency Medicine Assistant Professor of Emergency Medicine Clerkship Director Cooper Longitudinal Integrated Clerkship Cooper Medical School of Rowan University Cooper University Hospital Camden New Jersey USA cooperhealth.org

**Keywords:** acute pain, emergency department, fentanyl, intranasal analgesia, ketamine, meta-analysis, pain management, pediatric emergency medicine, randomized controlled trials, systematic review

## Abstract

**Background:**

Pain is one of the most common reasons for presentation to pediatric emergency departments, yet timely and effective analgesia remains inconsistently delivered. Intranasal (IN) analgesics offer a noninvasive alternative to injectable routes, but their comparative effectiveness, safety, and acceptability across agents and routes remain incompletely defined.

**Methods:**

We conducted a systematic review and network meta‐analysis of randomized controlled trials evaluating IN analgesics for acute pain management in children aged 0–18 years presenting to emergency or prehospital settings. Eligible trials compared IN agents with alternative IN formulations, intravenous, intramuscular, oral analgesics, or placebo. Pain reduction measured using validated pediatric pain scales at predefined timepoints was the primary outcome. Secondary outcomes included need for rescue analgesia, adverse events, acceptability, and emergency department length of stay.

**Findings:**

Fifteen randomized controlled trials involving 1388 pediatric patients were included. Across all evaluated timepoints, including the primary outcome and assessments at 10, 15, 20, 30, and 60 min, no IN or intravenous analgesic demonstrated statistically superior pain reduction compared with IN fentanyl. Requirements for rescue analgesia were similarly comparable across treatments. IN ketamine was associated with a significantly higher risk of adverse events compared with standard‐dose IN fentanyl, although events were predominantly mild and transient. Acceptability among patients, caregivers, and providers was high across IN agents, and available data indicated no clinically meaningful differences in emergency department length of stay.

## 1. Introduction

In pediatric emergency departments (EDs), pain is among the most common presenting symptoms. Estimates indicate that more than half of pediatric ED visits involve clinically significant pain [1, 2]. Despite this, optimal pain management remains a challenge. Multiple studies document delayed treatment, inconsistent assessment practices, and a substantial proportion of children being discharged while still experiencing moderate to severe pain [3, 4].

Traditional analgesic administration in acute care settings relies mainly on injectable routes. While widely used, these routes have several challenges in children who often have smaller and more difficult venous access, heightened anxiety, and greater sensitivity to needle‐based procedures. Although the overall success rate of intravenous administration is 95.7%, the first‐attempt success rate is only 53%, indicating that many children require multiple attempts, which can be painful and distressing [5]. Qualitative research among emergency medical services (EMS) providers has identified “IV line insertion pain” and difficulty obtaining IV access as major barriers to administering analgesia to children [6]. Intramuscular (IM) analgesia is similarly problematic; it is often discouraged in children because injections are painful and their onset of action can be unpredictable [7].

Given these limitations, there has been growing interest in noninvasive, rapid‐onset alternatives that can bypass the delays and distress associated with injectable routes. Intranasal (IN) analgesia has emerged as a promising option. Several pharmacologic agents, including fentanyl, ketamine, midazolam, and diamorphine, have been successfully delivered intranasally in pediatric settings. These agents are used across a range of painful conditions such as fractures, burns, and laceration‐related injuries [8], demonstrating an effective, safe, and favorable profile compared with traditional injectable routes. Multiple observational and interventional studies have reported analgesic onset within minutes, often faster than what is achievable with routes requiring IV placement [8, 9]. Beyond rapidity, IN medications may enhance the patient experience by minimizing procedural pain, reducing caregiver anxiety, and promoting greater cooperation during assessment and treatment [10].

Despite the expanding body of literature, the evidence base remains fragmented. Studies vary widely in patient age ranges, dosing regimens, analgesic agents, comparators, outcome measures, and clinical settings [11]. This variability makes it challenging for clinicians and guideline developers to determine the optimal IN agent, the most appropriate dosing strategies, and the clinical scenarios in which IN analgesia offers the greatest benefit over traditional routes. Furthermore, concerns regarding adverse effects, bioavailability differences, and variations in device delivery techniques underscore the need for a comprehensive analysis of existing randomized evidence.

Taken together, these gaps highlight the importance of synthesizing high‐quality comparative data to better understand the efficacy, onset of action, safety profile, and patient‐centered outcomes of IN analgesia in children presenting to the ED with acute pain. A systematic review and meta‐analysis of randomized controlled trials (RCTs) is therefore warranted to guide the integration of IN analgesia into evidence‐based pediatric emergency pain management protocols.

## 2. Methods

### 2.1. Protocol and Reporting

This systematic review was conducted based on a preoutlined protocol registered in the PROSPERO database (Registration number: CRD420251166487). The review adhered to the Cochrane Collaboration Guidelines [12] and the Preferred Reporting Items for Systematic Reviews and Meta‐Analyses (PRISMA) [13, 14]. Every step of the review process was planned to minimize bias and provide clinically meaningful results regarding the efficacy and safety of IN analgesia compared with other analgesic routes or placebo in children presenting with acute pain to the ED.

### 2.2. Eligibility Criteria

#### 2.2.1. Types of Studies

We included RCTs published in peer‐reviewed journals. Quasi‐randomized studies, observational studies, case series, case reports, conference abstracts lacking sufficient data, and noncomparative trials were excluded.

#### 2.2.2. Population

Participants aged 0–18 years presenting to an ED or prehospital emergency setting with acute painful conditions (e.g., fractures, burns, soft‐tissue injuries, and procedural pain) were included. One included study [15] enrolled patients up to 20 years of age; it was retained in the analysis due to its high relevance to the research question and the minimal deviation from the intended pediatric population.

#### 2.2.3. Interventions

IN analgesic medications administered for acute pain management. Accepted IN agents included, but were not limited to: fentanyl, ketamine, midazolam (when administered for analgesic or anxiolytic co‐analgesic effect), and diamorphine.

#### 2.2.4. Comparators

Eligible comparators included: intravenous, IM, or oral analgesics, alternative IN agents, and placebo or no analgesic intervention.

#### 2.2.5. Outcomes

*Primary outcome:* Pain reduction measured using validated pain scales (e.g., VAS, FPS‐R, and NRS) at the earliest available standardized timepoint (e.g., primary timepoint, 10, 15, 20, 30, 60 min). *Secondary outcomes:* Onset of analgesia, need for rescue analgesia, adverse events (e.g., respiratory depression, nausea, vomiting, dysphoria, and sedation), length of stay (LOS), and acceptability.

We acknowledge that the primary assessment timepoints varied across studies, reflecting differences in the pharmacokinetic properties of individual agents and clinical trial design. For agents with rapid onset, such as fentanyl (peak plasma concentration ∼7–10 min), earlier timepoints (e.g., 10–15 min) may be most informative, whereas for agents with slower onset or those requiring nasal absorption, later timepoints (e.g., 30–60 min) may better capture the peak effect. This variability is addressed in our analysis by evaluating pain reduction at multiple standardized timepoints (10, 15, 20, 30, and 60 min), allowing for a comprehensive assessment across different pharmacokinetic profiles.

### 2.3. Information Sources and Search Strategy

We conducted a comprehensive literature search in MEDLINE (via PubMed), Embase, Cochrane Central Register of Controlled Trials (CENTRAL), Web of Science, and ClinicalTrials.gov and WHO ICTRP for ongoing or unpublished trials. The search strategy combined keywords and MeSH terms related to IN administration, analgesia, pain, pediatrics, and ED. No restrictions were applied regarding language or publication years. Reference lists of included studies and relevant reviews were manually screened for additional eligible trials. Corresponding authors of key studies were contacted when more information or clarification was required.

### 2.4. Study Selection

Retrieved records were imported into a citation manager, and duplicates were removed. Two reviewers independently screened titles and abstracts for potential eligibility. Full texts of shortlisted articles were assessed against inclusion criteria. Discrepancies were resolved through consultation with a third reviewer. The study selection process is presented in the PRISMA flow diagram (Figure 1).

**FIGURE 1:**
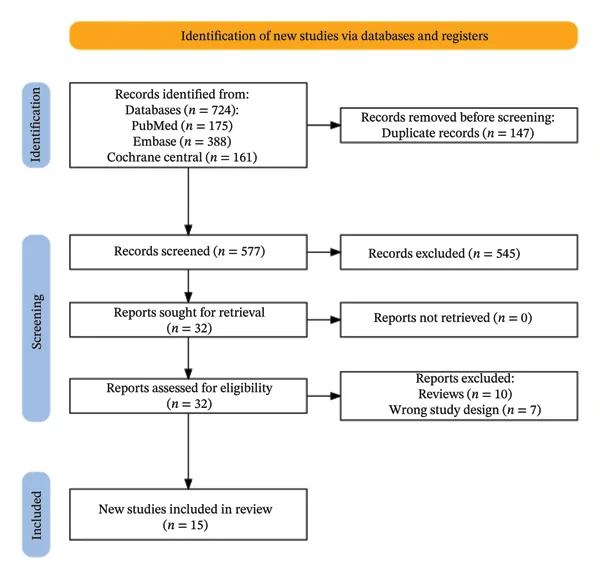
PRISMA flow diagram. Flowchart illustrating the systematic process of study identification, screening, eligibility assessment, and inclusion for the systematic review and network meta‐analysis of intranasal analgesia for acute pain in pediatric emergency departments.

### 2.5. Data Extraction

Data were extracted independently by two reviewers using a standardized and piloted form. Extracted data included:

• Study characteristics (authors, year, country, setting, and design)
• Participant demographics (age, sample size, and pain condition)
• Intervention details (agent, dose, and route of administration)
• Comparator characteristics
• Outcome measures and timepoints
• Adverse events and LOS

### 2.6. Quality Assessment

The Cochrane risk of bias 2 (RoB 2) tool was used for quality assessment [16]. The risk of bias for each study was assessed independently by two reviewers.

### 2.7. Acceptability Assessment

Acceptability was assessed using various measures across included studies. Parent/caregiver‐reported acceptability was typically evaluated using Likert‐scale questionnaires or binary (acceptable/not acceptable) assessments administered following the procedure or at discharge. Provider‐reported acceptability (nurses and/or physicians) was similarly assessed using structured questionnaires or rating scales. In the studies by Nikula et al. [17, 18], acceptability was measured using a 5‐point Likert scale. Kendall et al. [19] assessed acceptability using binary responses to the question ‘Was the method acceptable?’ administered separately to parents and providers. We defined acceptability as the proportion of respondents rating the intervention as acceptable (including responses of ‘acceptable’ or ratings ≥ 4 on 5‐point scales).

### 2.8. Data Synthesis

Data were pooled using a random‐effects meta‐analysis (DerSimonian–Laird method). Continuous outcomes were expressed as standardized mean differences (SMDs) with 95% confidence intervals (CIs), and dichotomous outcomes as risk ratios (RRs) with 95% CIs. Statistical heterogeneity was assessed using the I^2^ statistic. Subgroup analyses were performed by type of IN agent, age group, and pain etiology. Consistent trends and patterns observed across studies were highlighted in the synthesis.

## 3. Results

### 3.1. Selection Process

The PRISMA flow diagram (Figure 1) details the study selection process.

### 3.2. Study Characteristics

A total of 15 RCTs, including 1388 pediatric patients, were included in our review [8, 15, 17–29]. The included trials were conducted in multiple countries, including Sweden, Australia, Canada, and the United States of America (USA). The most representative country was the USA (6/16; 37.5%). Patients presented to the ED with various complaints, including suspected limb fractures, burns, lacerations, and migraine headaches. Mean ages ranged from 1.8 to 14.0 years. One study [15] included participants up to 20 years of age, but its inclusion was deemed appropriate as the core population and clinical context were highly pertinent to the pediatric review. All trials evaluated an IN agent compared with another IN drug, a different route of administration, or a placebo. The IN agents used across studies included fentanyl, ketamine, dexmedetomidine, esketamine, diamorphine, ketorolac, and lidocaine. Table 1 presents a summary of the characteristics of the included trials. Table 2 summarizes the baseline characteristics of the study population, including age, weight, sex, and type of injury.

**TABLE 1:** Characteristics of the 15 included randomized controlled trials evaluating intranasal analgesia for acute pain in pediatric emergency departments.

Study	Year	Country	Setting	PED vs mixed ED	Study design/phase	Sample size	Study period	Inclusion criteria	Exclusion criteria	Conclusion
Nikula et al. [17]–ESK vs DEX	2024	Sweden	Astrid Lindgren Children’s Hospital, Sweden	PED	Double‐blind, randomized superiority trial	30	July 2017 –Oct 2019	1–3 years, laceration or burn requiring PSA	ASA ≥ III, respiratory infection, neurological symptoms, hypersensitivity, insufficient Swedish understanding	Underpowered; no significant difference, but IN dexmedetomidine provided effective analgesia and sedation
Nikula et al. [18] – NO vs DEX	2024	Sweden	Astrid Lindgren Children’s Hospital, Sweden	PED	Open‐label, randomized noninferiority trial	148	Aug 2017 –Sep 2020	3–15 years, extremity fracture/luxation or burn requiring PSA	ASA ≥ III, respiratory/ear infection, vomiting, hypersensitivity, cardiac/neurological issues	IN dexmedetomidine noninferior to nitrous oxide for analgesia during painful procedures
Borland et al. [24]	2007	Australia	Princess Margaret Hospital for Children, Australia	PED	Double‐blind, randomized controlled trial	67	Sep 2001 –Jan 2005	7–15 years, clinically deformed closed long‐bone fracture	Narcotic within 4h, head injury, opiate allergy, nasal blockage, inability to pain score	Intranasal fentanyl equivalent to IV morphine for acute fracture pain
Borland et al. [25]	2011	Australia	Princess Margaret Hospital for Children, Australia	PED	Double‐blind, randomized controlled trial	189	Feb 2009 –Feb 2010	3–15 years, clinically deformed closed long‐bone fracture	Opiate within 4h, head injury, allergy, nasal blockage, inability to pain score, compound fracture	Standard and high‐concentration fentanyl are equivalent in pain reduction
Fein et al. [15]	2016	USA	Children’s Hospital at Montefiore, Bronx, NY	PED	Randomized, double‐blind, placebo‐controlled trial	49	Dec 2011 –Jan 2015	3–20 years, SCD, VOC pain score ≥ 6, not on daily opiates	Allergy to fentanyl, daily opiates, pregnancy, hypotension, O_2_ sat ≤ 92%, etc.	INF reduced pain more than placebo at 20 min; no serious adverse events
Frey et al. [8]	2019	USA	Cincinnati Children’s Hospital Medical Center, OH	PED	Double‐blind, randomized, active‐control, noninferiority trial	90	Mar 2016 – Feb 2017	8–17 years, extremity injury, VAS > 35 mm	Nasal trauma, opioid allergy, psychosis, non‐English speaking	Ketamine noninferior to fentanyl; more adverse events but all minor and transient
Graudins et al. [20]	2015	Australia	Monash Health hospitals, Australia	Mixed ED	Randomized, double‐blind, controlled trial	73	Nov 2012 –Nov 2013	3–13 years, limb injury, pain > 6/10, weight < 50 kg	Aberrant nasal anatomy, prior intranasal/opioid analgesia, allergy to study drugs	Similar pain reduction; ketamine had more minor adverse events
Ruffin et al. [29]	2022	USA	Arnold Palmer Hospital for Children, Orlando, FL	PED	Prospective, nonblinded, randomized controlled noninferiority trial	34	Aug 2018 –Mar 2019	6 months–18 years, painful infectious mouth lesions, poor oral intake	Severe dehydration, maxillofacial abnormalities, allergy to study drugs	INF as safe and effective as oral analgesic for pain and hydration
Kendall et al. [19]	2001	UK	8 UK Emergency Departments	Mixed ED	Multicentre RCT	404	Jul 1997– Sep 1999	Age 3–16 years; clinical fracture of an upper or lower limb	Not accompanied by parent/guardian; head injury; need for immediate IV access; blocked nose/URTI; learning difficulties; blindness; previous participation; recent opioids; contraindications to diamorphine/morphine	Nasal diamorphine spray provides faster onset of pain relief and is better tolerated and more acceptable than IM morphine. It should be the preferred method.
Tsze et al. [27]	2022	USA	Single US tertiary‐care children’s hospital ED	PED	Randomized, double‐blind, noninferiority clinical trial	56	Jun 2015–Mar 2021	Age 8–17 years; migraine headache; moderate to severe pain (≥ 4/10); required parenteral analgesics	Contraindications to ketorolac; recent NSAID; IN obstruction; inability to complete self‐reports; history of intracranial surgery/abnormalities; chronic pain conditions; critical illness; frequent headache medication use	Intranasal ketorolac was noninferior to intravenous ketorolac for reducing migraine pain at 60 min.
Maki et al. [21]	2022	Canada	Single Canadian Paediatric ED	PED	Pilot RCT (Double‐blind)	32	Jul 2019– Dec 2020	Age 7–16 years; migraine or posttraumatic headache with migraine features; VNRS pain score ≥ 4 after first‐line therapy	Trauma/seizure in last 24h; known seizure disorder; suspected intracranial pathology; fever/meningismus; lidocaine allergy; cardiac dysrhythmia; inability to assent	The pilot determined a sample size of 134 is needed for a future superiority trial. The protocol was feasible and safe.
Quinn et al. [23]	2018	USA	Single US teaching hospital	Mixed ED	Prospective, randomized, double‐blind, noninferiority study	22	May 2015–Nov 2016	Age 3–17 years; weight < 64 kg; acute moderate to severe pain (≥ 6/10) requiring opioid analgesia	Facial trauma/nasal anatomy abnormality; circulatory insufficiency; developmental delay; head trauma/altered consciousness; allergy to fentanyl/ketamine; inability to provide pain scale; recent opioids	Insufficient power to prove noninferiority of IN ketamine. IN ketamine had a slower onset (at 10 min) and higher rates of sedation and dizziness than IN fentanyl.
Ray et al. [26]	2002	India	Calcutta, India	Mixed ED	Prospective randomized controlled trial	32	Not specified	4–8 years, ASA I/II, elective surgery, Hannallah pain score ≥ 4 postop	Nasal infection/pathology	Intranasal fentanyl is a good alternative to IV fentanyl for postoperative analgesia in children.
Reynolds et al. [22]	2017	USA	Levine Children’s Hospital ED, Charlotte, North Carolina, USA	PED	Randomized controlled feasibility trial	87	Dec 2015 –Nov 2016	4–17 years, suspected isolated extremity fracture, pain score ≥ 4 (4–10 yrs) or ≥ 3 (11–17 yrs)	GCS < 15, allergy to ketamine/fentanyl, pregnancy, hypotension, weight > 70 kg, prior opioids, multiple injuries, nonverbal, nasal anatomy issues	Intranasal ketamine is feasible, has more minor side effects, and similar pain relief to intranasal fentanyl. Supports larger noninferiority trial.
Wilson et al. [28]	1997	UK	Bristol Frenchay Hospital, Bristol, UK	Mixed ED	Prospective randomized clinical trial	52	Jan–Sep 1995	3–16 years, clinically suspected limb fracture	Head injury, nasal obstruction, need for immediate IV access	Intranasal diamorphine is as effective as IM morphine, safer, and more acceptable to parents and staff.

*Note:* INF, intranasal fentanyl; IV, intravenous; IN, intranasal; IM, intramuscular.

Abbreviations: ASA, American Society of Anesthesiologists; ED, emergency department; GCS, glasgow coma scale; PED, pediatric emergency department; PSA, procedural sedation and analgesia; RCT, randomized controlled trial; SCD, sickle cell disease; VAS, visual analog scale; VNRS, verbal numeric rating scale; VOC, vaso‐occlusive crisis.

**TABLE 2:** Baseline demographic and clinical characteristics of the pediatric study populations from the included randomized controlled trials.

Study	Route	Drug arm	Sample size	Age (years)		Male		Weight		Procedure pain	Type of injury		Local anesthesia used (Y or N)	NSAID coadmin (Y or N)	Pre ED‐paracetamol (Y or N)
				Mean	SD	*n*	%	Mean	SD		*n*	%			
Nikula et al. [17]–ESK vs DEX	Intranasal	Dexmedetomidine	15	2.2		8	53.3	12.9		Laceration/Burn	12	80.0	Y	N	Y
	Intranasal	Esketamine	15	1.9		11	73.3	12.4		Laceration/Burn	15	100.0	Y	N	Y

Nikula et al. [18]–NO vs DEX	Intranasal	Dexmedetomidine	74	9.36	3.93	44	59.5	33.93	16.25	Fracture/Luxation/Burn	73	98.6	Y	N	Y
	Inhalation	Nitrous Oxide	74	9.13	4.0	40	54.1	33.96	15.19	Fracture/Luxation/Burn	72	97.3	Y	N	Y

Borland et al. [24]–INF vs IV morphine	Intranasal	Fentanyl	33	11.1				45.7		Fracture	26	78.8	N	N	N
	Intravenous	Morphine	34	10.7				41.9		Fracture	27	79.4	N	N	N

Borland et al. [25]–INF (standard)	Intranasal	Fentanyl (Standard)	98	9.1	3.535	59	60.2	35.1	14.899	Fracture	60	61.2	N	Y	N
	Intranasal	Fentanyl (High Concentration)	91	8.8	3.4	60	65.9	34.0	15.33	Fracture	58	63.7	N	Y	N

Fein et al. [15]	Intranasal	Fentanyl	24	10.6	5.3	11	46.0	34.83	22.459	VOC (Sickle Cell)	24	100.0	N	Y	N
	Intranasal	Placebo	25	12.5	5.1	19	76.0	48.0	32.23	VOC (Sickle Cell)	25	100.0	N	Y	N

Frey et al. [8]	Intranasal	Ketamine	44	11.8	2.6	26	59.0	45.8	14.4	Extremity Injury	44	100.0	N	N	N
	Intranasal	Fentanyl	42	12.2	2.3	31	74.0	50.8	22.8	Extremity Injury	42	100.0	N	N	N

Graudins et al. [20]	Intranasal	Ketamine	36	7.5	2.7	22	61.0			Limb Injury	36	100.0	N	Y	Allowed
	Intranasal	Fentanyl	37	8.66	3.85	24	65.0			Limb Injury	37	100.0	N	Y	Allowed

Ruffin et al. [29]	Intranasal	Fentanyl	17	3.1		7	41.0	15.2		Infectious Mouth Lesions	17	100.0	N	N	N
	Oral	Hydrocodone/Acetaminophen	17	1.8		10	59.0	12.5		Infectious Mouth Lesions	17	100.0	N	N	N

Kendall et al. [19]	Intranasal	Diamorphine	207	9.6	3.416	136	66.0	33.9	13.2	Fracture	204	100.0	N	N	N
	Intramuscular	Morphine	203	9.0	3.225	138	68.0	32.2	13.1	Fracture	200	100.0	N	N	N

Tsze et al. [27]	Intranasal	Ketorolac	27	13.66	3.91	10	37.0	57.7	16.9	Migraine	27	100.0	N	N	N
	Intravenous	Ketorolac	29	14.0	3.989	8	27.6	63.7	26.8	Migraine	29	100.0	N	N	N

Maki et al. [21]	Intranasal	Lidocaine	17	11.8	3.36	9	53.0			Migraine/PTH	17	100.0	N	N	N
	Intranasal	Placebo	15	12.7	2.49	4	27.0			Migraine/PTH	15	100.0	N	N	N

Quinn et al. [23]	Intranasal	Fentanyl	11	9.58	2.92	8	73.0			MSK/Abdominal	11	100.0	N	?	?
	Intranasal	Ketamine	11	9.77	2.51	10	91.0			MSK/Abdominal	11	100.0	N	?	?

Ray et al. [26]	Intranasal	Fentanyl	16	5.6	1.76			16.26	4.65	Postoperative	16	100.0	N	N	N
	Intravenous	Fentanyl	16	5.8	2.07			16.95	4.5	Postoperative	16	100.0	N	N	N

Reynolds et al. [22]	Intranasal	Ketamine	43			26	61.0			Extremity Fracture	43	100.0	N	Y	N
	Intranasal	Fentanyl	44			28	64.0			Extremity Fracture	44	100.0	N	Y	N

Wilson et al. [28]	Intranasal	Diamorphine	30	7.4						Fracture	30	100.0	N	N	N
	Intramuscular	Morphine	22	7.9						Fracture	22	100.0	N	N	N

*Note:* Data are presented by treatment arm for each study. MSK, musculoskeletal; Pre‐ED, pre‐emergency department.

Abbreviations: NSAID, nonsteroidal anti‐inflammatory drug; PTH, posttraumatic headache; SD, standard deviation.

### 3.3. Quality Assessment

Risk of bias for the 15 included RCTs was assessed using the ROB‐2 tool. One study was judged to have a high risk of bias due to missing outcome data and inappropriate measurement tools. Six studies had some concerns, while the remaining eight were rated as low risk. A summary of the quality assessment is presented in Figure 2, and detailed judgments for each study are provided in Supporting Figure S1.

**FIGURE 2:**
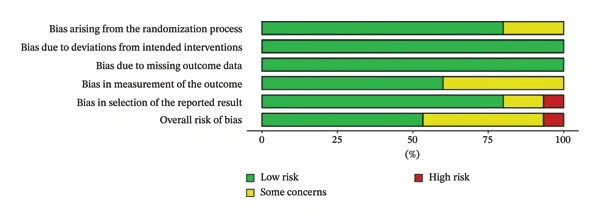
Risk of bias summary. Graphical summary of the methodological quality assessment for the 15 included randomized controlled trials using the Cochrane risk of bias (RoB 2) tool. Judgments (low risk, some concerns, and high risk) are displayed across five domains and as an overall assessment for all studies.

### 3.4. Outcomes

#### 3.4.1. Pain Reduction Outcomes

##### 3.4.1.1. Pain Reduction at the Primary Timepoint

Six studies reported the pain reduction at the primary timepoint, comprising 339 patients [8, 15, 20–23]. The treatment comparison groups consisted of three interventions and a placebo, forming a connected network of three designs and six pairwise comparisons (Figure 3A). In the common‐effects model (Figure 4A), IN ketamine (MD = 0.36, 95% CI: −0.21–0.92; *p* = 0.21), IN lidocaine (MD = −0.51, 95% CI: −3.11–2.10; *p* = 0.70), and placebo (MD = −1.17, 95% CI: −2.43–0.10; *p* = 0.07) did not show a significant effect compared with IN fentanyl (Table 3). No statistically significant indirect effects were observed in any contrast, and SIDE analyses indicated no disagreement between direct and indirect evidence. Heterogeneity within pairwise comparisons was negligible (tau^2^ = 0; I^2^ = 0%). SUCRA ranking suggested that IN ketamine had the highest probability of reducing pain among the treatments (83%), followed by IN fentanyl (63%), IN lidocaine (43%), and placebo (11%). Despite these rankings, the absence of statistically significant differences indicates that these results should be interpreted cautiously.

**FIGURE 3:**
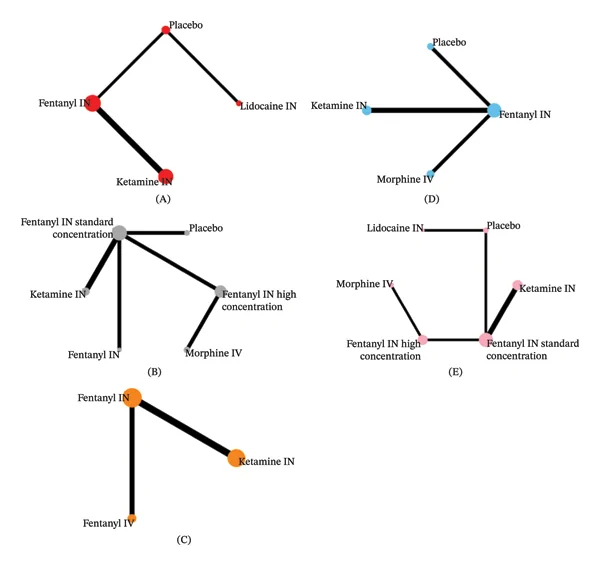
Network geometry of comparisons for pain reduction network plots displaying the available direct comparisons between interventions for the outcome of pain reduction at each analyzed timepoint. (A) Primary timepoint. (B) 10 min. (C) 15 min. (D) 20 min. (E) 30 min.

**FIGURE 4:**
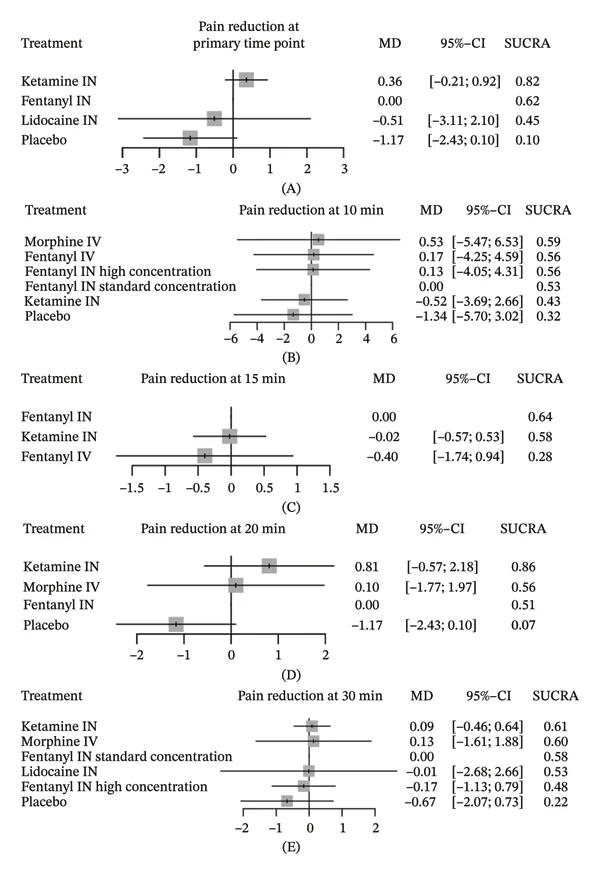
Forest plots for pain reduction at different timepoints forest plots displaying pooled mean differences (MD) with 95% confidence intervals (CI) for pain reduction, with intranasal fentanyl (standard concentration, 50 mcg/mL) as the reference treatment. (A) Primary timepoint. (B) 10 min. (C) 15 min. (D) 20 min. (E) 30 min.

**TABLE 3:** League table of network meta‐analysis results.

**Pain reduction at primary timepoint MD (95% CI)**						
	**Fentanyl IN**	**Ketamine IN**	**Lidocaine IN**		**Placebo**	

Fentanyl IN		−0.36 (−0.92–0.21)	0.51 (−2.10–3.11)		1.17 (−0.10–2.43)	
Ketamine IN	−0.36 (−0.92–0.21)		0.86 (−1.80–3.53)		**1.52 (0.14–2.91)**	
Lidocaine IN	0.51 (−2.10–3.11)	0.86 (−1.80–3.53)			0.66 (−1.61–2.93)	
Placebo	1.17 (−0.10–2.43)	**1.52 (0.14–2.91)**	0.66 (−1.61–2.93)			

**Pain reduction at 10 min MD (95% CI)**						
	**Fentanyl IN high concentration**	**Fentanyl IN standard concentration**	**Fentanyl IV**	**Ketamine IN**	**Morphine IV**	**Placebo**

Fentanyl IN high concentration		0.13 (−4.05–4.31)	−0.04 (−6.12–6.04)	0.65 (−4.61–5.90)	−0.40 (−4.70–3.90)	1.47 (−4.57–7.51)
Fentanyl IN standard concentration	0.13 (−4.05–4.31)		−0.17 (−4.59–4.25)	0.52 (−2.66–3.69)	−0.53 (−6.53–5.47)	1.34 (−3.02–5.70)
Fentanyl IV	−0.04 (−6.12–6.04)	−0.17 (−4.59–4.25)		0.69 (−4.76–6.13)	−0.36 (−7.81–7.09)	1.51 (−4.70–7.72)
Ketamine IN	0.65 (−4.61–5.90)	0.52 (−2.66–3.69)	0.69 (−4.76–6.13)		−1.05 (−7.83–5.74)	0.82 (−4.57–6.22)
Morphine IV	−0.40 (−4.70–3.90)	−0.53 (−6.53–5.47)	−0.36 (−7.81–7.09)	−1.05 (−7.83–5.74)		1.87 (−5.54–9.28)
Placebo	1.47 (−4.57–7.51)	1.34 (−3.02–5.70)	1.51 (−4.70–7.72)	0.82 (−4.57–6.22)	1.87 (−5.54–9.28)	

**Pain reduction at 15 min MD (95% CI)**						
	**Fentanyl IN**	**Fentanyl IV**			**Ketamine IN**	

Fentanyl IN		0.40 (−0.94–1.74)			0.02 (−0.53–0.57)	
Fentanyl IV	0.40 (−0.94–1.74)				−0.38 (−1.82–1.07)	
Ketamine IN	0.02 (−0.53–0.57)	−0.38 (−1.82–1.07)				

**Pain reduction at 20 min MD (95% CI)**						
	**Fentanyl IN**	**Ketamine IN**	**Morphine IV**		**Placebo**	

Fentanyl IN		−0.81 (−2.18–0.57)	−0.10 (−1.97–1.77)		1.17 (−0.10–2.43)	
Ketamine IN	−0.81 (−2.18–0.57)		0.71 (−1.61–3.03)		**1.97 (0.11–3.84)**	
Morphine IV	−0.10 (−1.97–1.77)	0.71 (−1.61–3.03)			1.27 (−0.99–3.52)	
Placebo	1.17 (−0.10–2.43)	**1.97 (0.11–3.84)**	1.27 (−0.99–3.52)			

**Pain reduction at 30 min MD (95% CI)**						
	**Fentanyl IN high concentration**	**Fentanyl IN standard concentration**	**Ketamine IN**	**Lidocaine IN**	**Morphine IV**	**Placebo**

Fentanyl IN high concentration		−0.17 (−1.23–0.89)	−0.27 (−1.50–0.95)	−0.16 (−3.10–2.79)	−0.30 (−1.83–1.23)	0.50 (−1.31–2.32)
Fentanyl IN standard concentration	−0.17 (−1.23–0.89)		−0.11 (−0.72–0.51)	0.01 (−2.74–2.76)	−0.13 (−1.99–1.73)	0.67 (−0.80–2.14)
Ketamine IN	−0.27 (−1.50–0.95)	−0.11 (−0.72–0.51)		0.12 (−2.70–2.93)	−0.03 (−1.98–1.93)	0.78 (−0.82–2.37)
Lidocaine IN	−0.16 (−3.10–2.79)	0.01 (−2.74–2.76)	0.12 (−2.70–2.93)		−0.14 (−3.46–3.17)	0.66 (−1.66–2.98)
Morphine IV	−0.30 (−1.83–1.23)	−0.13 (−1.99–1.73)	−0.03 (−1.98–1.93)	−0.14 (−3.46–3.17)		0.80 (−1.57–3.18)
Placebo	0.50 (−1.31–2.32)	0.67 (−0.80–2.14)	0.78 (−0.82–2.37)	0.66 (−1.66–2.98)	0.80 (−1.57–3.18)	

**Rescue medications RR (95% CI)**						
	**Fentanyl IN**	**Ketamine IN**			**Morphine IV**	

Fentanyl IN		1.24 (0.75–2.05)			2.06 (0.20–21.65)	
Ketamine IN	1.24 (0.75–2.05)				1.66 (0.15–18.38)	
Morphine IV	2.06 (0.20–21.65)	1.66 (0.15–18.38)				

**All adverse events RR (95% CI)**						
	**Fentanyl IN high concentration**	**Fentanyl IN standard concentration**	**Fentanyl IV**	**Ketamine IN**	**Morphine IV**	

Fentanyl IN high concentration		1.45 (0.92–2.29)	2.18 (0.39–12.06)	0.75 (0.46–1.24)	2.06 (0.40–10.50)	
Fentanyl IN standard concentration	1.45 (0.92–2.29)		1.50 (0.29–7.81)	**0.52 (0.43–0.63)**	1.42 (0.26–7.71)	
Fentanyl IV	2.18 (0.39–12.06)	1.50 (0.29–7.81)		0.35 (0.07–1.83)	0.95 (0.09–10.05)	
Ketamine IN	0.75 (0.46–1.24)	**0.52 (0.43–0.63)**	0.35 (0.07–1.83)		2.73 (0.50–14.99)	
Morphine IV	2.06 (0.40–10.50)	1.42 (0.26–7.71)	0.95 (0.09–10.05)	2.73 (0.50–14.99)		

**Mild adverse events RR (95% CI)**						
	**Fentanyl IN**	**Hydrocodone/acetaminophen oral**	**Ketamine IN**		**Morphine IV**	

Fentanyl IN		0.69 (0.47–1.00)	0.60 (0.15–2.35)		2.06 (0.40–10.50)	
Hydrocodone/acetaminophen oral	0.69 (0.47–1.00)		0.87 (0.21–3.59)		3.00 (0.56–15.92)	
Ketamine IN	0.60 (0.15–2.35)	0.87 (0.21–3.59)			3.43 (0.41–28.74)	
Morphine IV	2.06 (0.40–10.50)	3.00 (0.56–15.92)	3.43 (0.41–28.74)			

**Acceptability (parents) RR (95% CI)**						
	**Dexmedetomidine IN**	**Esketamine IN**			**Nitrous oxide inhalation**	

Dexmedetomidine IN		1.17 (0.94–1.47)			0.93 (0.84–1.03)	
Esketamine IN	1.17 (0.94–1.47)				0.79 (0.62–1.01)	
Nitrous oxide inhalation	0.93 (0.84–1.03)	0.79 (0.62–1.01)				

**Acceptability (nurse/doctor/provider) RR (95% CI)**						
	**Dexmedetomidine IN**	**Esketamine IN**			**Nitrous oxide inhalation**	

Dexmedetomidine IN		1.33 (0.85–2.08)			0.92 (0.79–1.07)	
Esketamine IN	1.33 (0.85–2.08)				0.69 (0.43–1.11)	
Nitrous oxide inhalation	0.92 (0.79–1.07)	0.69 (0.43–1.11)				

*Note:* The table presents effect estimates (mean difference [MD] with 95% confidence interval [CI] for pain reduction; risk ratio [RR] with 95% CI for dichotomous outcomes) for all treatment comparisons at each assessed timepoint and for secondary outcomes. Significant results are highlighted in bold. Comparisons should be read from the row‐defining treatment to the column‐defining treatment. The league table shows the effect estimate of treatments in pain reduction at different timepoints and other secondary outcomes. IN, intranasal; IV, intravenous; IM, intramuscular.

Abbreviations: CI, confidence interval; MD, mean difference; RR, risk ratio.

##### 3.4.1.2. Pain Reduction at 10 Min

Six studies involving 445 patients across five treatments and a placebo, forming five designs and six pairwise comparisons (Figure 3B), reported pain reduction at the 10‐min timepoint [15, 22–26]. Significant heterogeneity was detected within the network (tau^2^ = 4.44, I^2^ = 84%). The random‐effects model did not demonstrate statistically significant differences between any of the arms and IN fentanyl at standard concentration (50 mcg/mL) (Figure 4B). The estimated mean differences were as follows: IN fentanyl at high concentration (150–300 mcg/mL) (MD = 0.13, 95% CI: −4.05–4.31; *p* = 0.95), intravenous fentanyl (MD = 0.17, 95% CI: −4.25–4.59; *p* = 0.94), IN ketamine (MD = −0.52, 95% CI: −3.69–2.66; *p* = 0.75), intravenous morphine (MD = 0.53, 95% CI: −5.47–6.53; *p* = 0.86), and placebo (MD = −1.34, 95% CI: −5.70–3.02; *p* = 0.55) (Table 3). No disagreements between direct and indirect evidence were detected through the SIDE analysis. The SUCRA ranking exhibited that intravenous morphine had the highest probability of reducing pain (60.9%), followed by intravenous fentanyl (55.8%), IN fentanyl at high concentration (150–300 mcg/mL) (55.4%), IN fentanyl at low concentration (54.0%), and IN ketamine (44.1%).

##### 3.4.1.3. Pain Reduction at 15 Min

Three studies comprising 187 patients reported pain reduction data at the 15‐min timepoint. The treatment comparison groups consisted of three interventions, forming a connected network of two designs and three pairwise comparisons (Figure 3C) [8, 20, 26]. In the common‐effects model (Figure 4C), comparisons with IN fentanyl yielded no statistically significant differences: intravenous fentanyl (MD = −0.40, 95% CI: −1.74–0.94; *p* = 0.56) and IN ketamine (MD = −0.02, 95% CI: −0.57–0.53; *p* = 0.94) (Table 3). Heterogeneity within pairwise comparisons and inconsistency were negligible (tau^2^ = 0; I^2^ = 0%). SIDE analysis showed no quantifiable disagreement between direct and indirect evidence. The SUCRA ranking suggested IN fentanyl as the most effective treatment (63.6%), followed by IN ketamine (58.3%), while intravenous fentanyl was the worst (28.1%).

##### 3.4.1.4. Pain Reduction at 20 Min

Four studies reported the reduction in pain at the 20‐min timepoint, comprising 221 patients [15, 22–24]. The treatment comparison groups consisted of three interventions and a placebo, forming a connected network of three designs and four pairwise comparisons (Figure 3D). In the common‐effects (Figure 4D), IN ketamine (MD = 0.81, 95% CI: −0.57–2.18; *p* = 0.25), intravenous morphine (MD = 0.10, 95% CI: −1.77–1.97; *p* = 0.92), and placebo (MD = −1.17, 95% CI: −2.43–0.10; *p* = 0.07) did not exhibit a significant effect when compared with IN fentanyl (Table 3). SIDE analyses indicated no disagreement between direct and indirect evidence. Heterogeneity within pairwise comparisons was negligible (tau^2^ = 0; I^2^ = 0%). SUCRA values suggested that IN ketamine had the highest probability of effectiveness (85.9%), followed by intravenous morphine (55.0%), IN fentanyl (51.9%), and placebo (7.2%).

##### 3.4.1.5. Pain Reduction at 30 Min

Eight studies with 592 observations documented data at the 30‐min timepoint [8, 15, 20–25]. The network consisted of five treatments and a placebo, forming five designs and eight pairwise comparisons (Figure 3E). Relative to IN fentanyl at standard concentration (50 mcg/mL), the common‐effects model did not demonstrate any statistically significant differences with any of the arms: IN fentanyl at high concentration (150‐300 mcg/mL) (MD = −0.17, 95% CI: −1.13–0.79; *p* = 0.73), IN ketamine (MD = 0.09, 95% CI: −0.46–0.64; *p* = 0.76), IN lidocaine (MD = −0.01, 95% CI: −2.68–2.66; *p* = 0.99), intravenous morphine (MD = 0.13, 95% CI: −1.61–1.88; *p* = 0.88), and placebo (MD = −0.67, 95% CI: −2.07–0.73; *p* = 0.35) (Figure 4E and Table 3). Heterogeneity within pairwise comparisons was low (tau^2^ = 0.05, I^2^ = 11.8%). SIDE analysis showed no evidence of disagreement between direct and indirect estimates. IN ketamine achieved the highest SUCRA ranking (62.0%), followed by intravenous morphine (58.7%), IN fentanyl at standard concentration (50 mcg/mL) (57.4%), IN lidocaine (53.4%), IN fentanyl at high concentration (150‐300 mcg/mL) (46.3%), and placebo (22.1%).

##### 3.4.1.6. Pain Reduction at 60 Min

IN fentanyl and ketamine were assessed at the 60‐min timepoint. The pooled analysis did not demonstrate a significant difference between both arms (MD = 0.01; 95% CI: −0.60–0.61; *p* = 0.98) [8, 20, 22, 23]. Between‐study variance was negligible (tau^2^ = 0; I^2^ = 0%) (Figure S2).

#### 3.4.2. Other Drugs Assessed Pain Reduction at Different Timepoints

Tsze et al., a single US tertiary‐care children’s hospital, compared IN and intravenous ketorolac across 59 patients and produced comparable reductions in pain intensity across all measured timepoints: 10 min (MD = 0.9, 95% CI: −0.4–2.2), at 30 min (MD = 0.8, 95% CI: −0.4–1.9), at 60 min (MD = 0.2, 95% CI: −0.9–1.3), and at 120 min (MD = 0.0, 95% CI: −1.3–1.2). Across all intervals, no statistically significant differences were reported between IN and intravenous ketorolac in terms of pain reduction [27].

Wilson et al., an RCT involving 52 pediatric patients with suspected limb fractures, compared IN diamorphine with IM morphine. The summed median pain scores were 9 and 8, respectively; however, the difference was not statistically significant (*p* = 0.4) [28].

#### 3.4.3. Need for Rescue Medications

Four studies contributed data on the need for rescue medications, including 314 participants, forming a network of three treatments and four pairwise comparisons (Figure 5A) [8, 20, 22, 24]. In the common‐effects model (Figure 6A), neither IN ketamine (RR = 0.81, 95% CI: 0.49 to 1.33; *p* = 0.40) nor intravenous morphine (RR = 0.49, 95% CI: 0.05 to 5.10; *p* = 0.55) demonstrated statistically significant differences when compared with IN fentanyl. Heterogeneity was low (tau^2^ = 0.06; I^2^ = 24.8%; *p* = 0.26) (Table 3). No inconsistency was detected between direct and indirect comparisons. Based on SUCRA rankings, intravenous morphine exhibited better findings than IN treatments (69%), followed by IN ketamine (57%), while IN fentanyl ranked last (24%). Through a direct meta‐analysis of IN diamorphine versus IM morphine, the analysis exhibited no significant difference between the two drugs (RR = 0.88; 95% CI: 0.38–2.05; *p* = 0.78) (Figure S3) [19, 28]. Between‐study variance was negligible (tau^2^ = 0; I^2^ = 0%).

**FIGURE 5:**
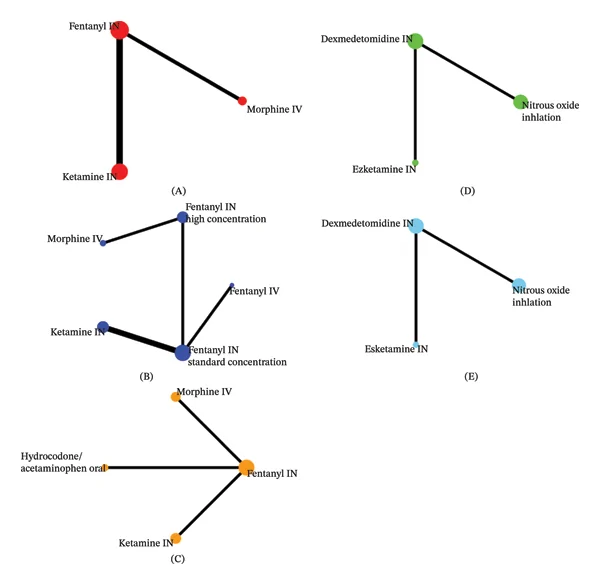
Network geometry of comparisons for secondary outcomes network plots displaying the available direct comparisons between interventions for key secondary outcomes. (A) Need for rescue analgesia. (B) Incidence of all adverse events. (C) Incidence of mild adverse events. (D) Parent‐reported acceptability. (E) Provider‐reported acceptability. Node size and line thickness are proportional to sample size and number of studies, respectively.

**FIGURE 6:**
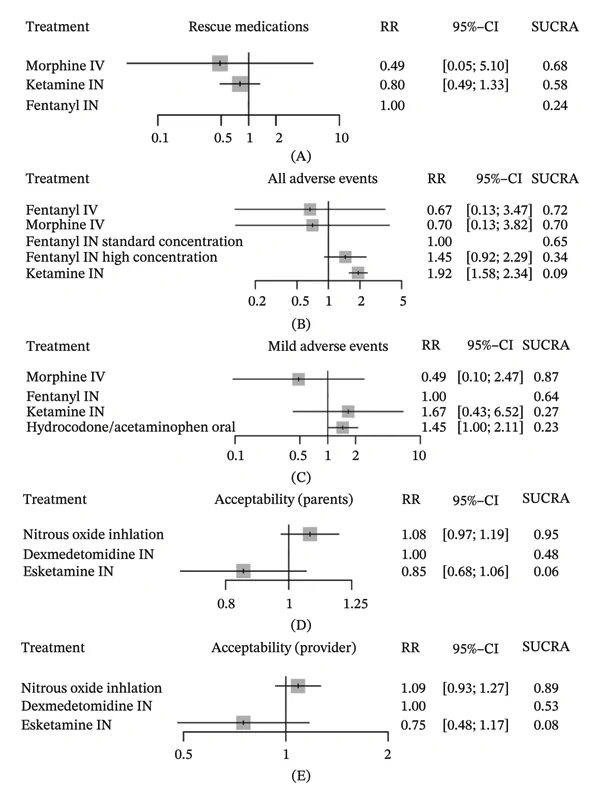
Forest plots for secondary outcomes. Forest plots displaying pooled risk ratios (RR) with 95% confidence intervals (CI) for secondary outcomes against their respective reference treatments. (A) Need for rescue analgesia (Ref: IN Fentanyl). (B) All adverse events (Ref: IN Fentanyl Std). (C) Mild adverse events (Ref: IN Fentanyl). (D) Parent acceptability (Ref: IN Dexmedetomidine). (E) Provider acceptability (Ref: IN Dexmedetomidine).

#### 3.4.4. Adverse Events

All adverse events data were collected from seven studies, involving 554 participants and forming a network of five treatments and seven pairwise comparisons (Figure 5B) [8, 20, 22–26]. In the common‐effects model (Figure 6B), IN ketamine was associated with a significantly higher risk of adverse events compared with IN fentanyl at standard concentration (50 mcg/mL) (RR = 1.92, 95% CI: 1.58–2.34; *p* < 0.0001). No statistically significant differences were shown for high concentration IN fentanyl (150‐300 mcg/mL) (RR = 1.45, 95% CI: 0.92–2.29; *p* = 0.11), intravenous fentanyl (RR = 0.67, 95% CI: 0.13–3.47; *p* = 0.63), intravenous morphine (RR = 0.70, 95% CI: 0.13–3.82; *p* = 0.68) when compared with IN fentanyl at standard concentration (50 mcg/mL). Heterogeneity within the network was low (tau^2^ = 0.015; I^2^ = 22.2%; *p* = 0.28) (Table 3). SIDE analyses revealed complete agreement between direct and indirect estimates. Ranking analyses demonstrated that intravenous fentanyl had the most favorable safety profile (74%), followed by intravenous morphine (70%) and IN fentanyl at standard concentration (50 mcg/mL) (65%). Ultimately, IN fentanyl at high concentration (150‐300 mcg/mL) and IN ketamine ranked markedly lower (34% and 7%, respectively).

In terms of the presence of mild adverse events, three studies involving 183 participants contributed to a network of four treatments and three pairwise comparisons (Figure 5C) [22, 24, 29]. Using a common‐effects model (Figure 6C), oral hydrocodone/acetaminophen demonstrated a significantly higher risk of mild adverse events compared with IN fentanyl (RR = 1.45, 95% CI: 1.00–2.11; *p* = 0.047). Neither IN ketamine (RR = 1.67, 95% CI: 0.43–6.52; *p* = 0.46) nor intravenous morphine (RR = 0.49, 95% CI: 0.10–2.47; *p* = 0.38) exhibited a statistically significant difference relative to IN fentanyl (Table 3). No heterogeneity or inconsistency could be quantified. Direct and indirect estimates were identical across all contrasts. The SUCRA values indicated that intravenous morphine had the most favorable profile (86%), followed by IN fentanyl (65%); however, ketamine and hydrocodone/acetaminophen ranked the worst (26% and 23%, respectively).

#### 3.4.5. Acceptability

Acceptability data for parents were derived from two studies, encompassing 173 participants and forming a network of three treatments with two pairwise comparisons (Figure 5D) [17, 18]. In the common‐effects model (Figure 6D), neither IN esketamine (RR = 0.85, 95% CI: 0.68–1.06; *p* = 0.16) nor inhaled nitrous oxide (RR = 1.08, 95% CI: 0.97–1.19; *p* = 0.15) demonstrated statistically significant differences when compared with IN dexmedetomidine (Table 3). No heterogeneity was quantified across the network. SIDE analyses indicated no disagreements between direct and indirect estimates. Pooled ranking analysis suggested that inhaled nitrous oxide had the highest acceptability (94%), followed by IN dexmedetomidine (49%), while the worst was IN esketamine (6%).

Similarly, provider‐reported acceptability was also collected from the same two studies, which involved 176 participants across three treatments and two pairwise comparisons (Figure 5E) [17, 18]. Under the common‐effects model (Figure 6E), IN esketamine and inhaled nitrous oxide did not demonstrate significant differences compared to IN dexmedetomidine (RR = 0.75, 95% CI: 0.48–1.17; *p* = 0.21 and RR = 1.09, 95% CI: 0.93–1.27; *p* = 0.29, respectively) (Table 3). No heterogeneity was quantified across the network. The SIDE analysis revealed no disagreement between direct and indirect comparisons. Ranking analyses summarized that inhaled nitrous oxide had the highest acceptability (90%), followed by IN dexmedetomidine (51%), while IN esketamine consistently ranked the lowest (8%).

Kendall et al., a multicenter RCT in 8 UK EDs compared IN diamorphine with IM morphine and demonstrated markedly superior provider acceptability with the IN route (199/203; 98% vs. 64/199; 32%; *p* < 0.0001). Parent‐reported acceptability at 30 min also favored the IN route (*p* < 0.0001), with 97% (186/192) rating the method acceptable compared with 72% (142/197) in the IM group [19].

Nikula et al. [17], a Swedish RCT comparing IN dexmedetomidine with inhaled nitrous oxide, found no significant difference in patient acceptability (*p* = 0.46). In contrast, Kendall et al. [19] compared IN diamorphine with IM morphine and reported markedly higher patient acceptability with the IN route (162/203; 79.8% vs. 17/199; 8.5%; *p* < 0.0001) [17].

#### 3.4.6. LOS

Maki et al. compared IN lidocaine with placebo in a Canadian pediatric ED (32 patients) and reported shorter lengths of stay in the IN lidocaine group (175.33 ± 84.07 min) compared with placebo (196.83 ± 118.59 min) [21]. Reynolds et al., a randomized controlled feasibility trial involving 87 patients, found that from ED presentation to sedation or discharge, the median time was 304 min (IQR 259–374) in the IN ketamine group and 327 min (IQR 263–408) in the IN fentanyl group [22].

## 4. Discussion

This network meta‐analysis, synthesizing data from 15 RCTs involving 1388 children, demonstrates that IN fentanyl, IN ketamine, and other IN or intravenous agents provide statistically comparable pain relief across all measured timepoints. At the primary timepoint, none of the agents significantly differed from IN fentanyl: ketamine (MD 0.36, 95% CI −0.21–0.92), lidocaine (MD −0.51, 95% CI −3.11–2.10), or placebo (MD −1.17, 95% CI −2.43–0.10). Across sequential assessments at 10, 15, 20, 30, and 60 min, no superior analgesic emerged over IN fentanyl, with all MDs crossing zero. Rescue medication requirements were similar between IN fentanyl and IN ketamine (RR 0.81, 95% CI 0.49–1.33) and IV morphine (RR 0.49, 95% CI 0.05–5.10).

IN ketamine demonstrated a significantly higher risk of adverse events compared with IN fentanyl (RR 1.92, 95% CI 1.58–2.34). Acceptability was high across IN agents with no significant differences. Collectively, these findings reinforce the clinical equivalence in analgesic efficacy but highlight a better safety profile for IN fentanyl.

Our pooled analysis consistently demonstrated no statistically significant differences in pain reduction between IN fentanyl and other analgesics across all time intervals (MDs from −1.34 to +0.53, all *p* > 0.05). These results align with contemporary pediatric trials showing noninferiority between IN agents. Frey et al. reported noninferiority of IN ketamine to IN fentanyl at 15 and 30 min in extremity injuries [8]. Similarly, Tsze et al. demonstrated no difference between IN and IV ketorolac at 10, 30, 60, and 120 min in pediatric migraine [27]. Differences across studies largely reflect disease heterogeneity, variable pain scales, and different mucosal absorption patterns.

Pharmacokinetic differences between IN agents warrant consideration in clinical decision‐making. IN fentanyl achieves peak plasma concentrations within approximately 7–10 min, offering the most rapid onset among the agents evaluated in this review, which aligns with the early convergence in pain scores observed at 10 and 15 min in our analysis [30]. Ketamine, while achieving comparable peak concentrations within a similar timeframe, may be associated with greater discomfort during administration due to its relatively acidic formulation (pH 3.5–5.5) [31]. This formulation‐related discomfort, though not systematically captured in the included trials, may impact patient acceptability, particularly in younger children. Dexmedetomidine and esketamine, while promising, have more limited pediatric pharmacokinetic data and fewer comparative trials, precluding definitive conclusions about their onset profiles relative to fentanyl and ketamine. Clinically, these findings confirm that IN analgesics, especially fentanyl and ketamine, provide rapid, reliable pain relief comparable to intravenous options, supporting their increasing adoption in ED protocols that favor minimal invasiveness and faster administration.

### 4.1. Dosing Considerations Across Included Studies

A comparison of dosing regimens across the included trials reveals important variability that may influence the interpretation of efficacy and safety outcomes. For IN fentanyl, doses ranged from 1.5 to 2.0 mcg/kg (standard concentration, 50 mcg/mL) to 3.0–4.5 mcg/kg (high concentration, 150‐300 mcg/mL), with bioavailability estimated at approximately 70%–80% compared with intravenous administration [30]. When comparing IN fentanyl to IV fentanyl, the doses used were generally pharmacologically comparable when accounting for this bioavailability difference. For example, Borland et al. [24] used IN fentanyl 1.5 mcg/kg compared with IV morphine 0.1 mg/kg, while the absolute doses of fentanyl in the Reynolds et al. [22] and Graudins et al. [20] studies were adjusted to account for the expected bioavailability of the IN route.

For IN ketamine, a consistent dose of 1 mg/kg was used across most studies [8, 20, 22, 23], with bioavailability estimated at approximately 45%–50% [31]. This dose was selected based on previous pharmacokinetic data and clinical experience to achieve comparable analgesic efficacy to standard‐dose IN fentanyl (1.5–2.0 mcg/kg). It is noteworthy that the higher dose of ketamine (1 mg/kg) relative to fentanyl (1.5–2.0 mcg/kg) reflects the drugs’ substantially different potencies and pharmacokinetic profiles.

Among other agents, IN lidocaine (4 mg/kg, 4% concentration) was used by Maki et al. [21], IN diamorphine (0.1 mg/kg) by Wilson et al. [28] and Kendall et al. [19], and IN ketorolac (15 mg) by Tsze et al. [27]. These doses were selected based on established safety profiles and prior efficacy data for each agent. For dexmedetomidine, Nikula et al. used 3 mcg/kg [17, 18], and esketamine was administered at 1 mg/kg [17].

The variability in dosing across studies, while clinically appropriate for each agent, complicates direct comparisons of efficacy and safety. When interpreting our network meta‐analysis results, it is important to recognize that IN fentanyl at standard concentration (50 mcg/mL, 1.5–2.0 mcg/kg) served as the reference treatment, and other agents were compared using doses that were considered clinically comparable by the original investigators. However, the absence of formal dose‐finding studies for some agents in the pediatric ED setting underscores the need for future research to optimize dosing strategies for individual agents and patient populations.

There were no significant differences in rescue medication requirement between IN ketamine (RR 0.81, 95% CI 0.49–1.33) or IV morphine (RR 0.49, 95% CI 0.05–5.10) when compared with IN fentanyl, with overall low heterogeneity in the pooled network. This finding is consistent with several rigorous randomized trials and systematic reviews. In the PRIME randomized clinical trial [8], IN ketamine and IN fentanyl produced similar requirements for rescue analgesia (relative risk for rescue 0.89; 95% CI 0.5–1.6), supporting the lack of a clinically important difference between these agents. The PICHFORK randomized trial [20] likewise reported comparable rescue‐analgesia needs for IN ketamine and IN fentanyl while documenting more frequent but largely minor adverse events with ketamine. Taken together, these external data corroborate our pooled estimates and suggest that observed variations in rescue medication rates across studies are more likely explained by protocol differences, thresholds for rescue and monitoring practices, and patient or procedure mix than by large inherent differences in single‐dose analgesic efficacy between IN fentanyl and IN ketamine [32]. Clinically, the convergence of evidence justifies treating IN fentanyl and IN ketamine as largely interchangeable first‐line single‐dose options for rapid analgesia in many pediatric emergency settings, with agent selection guided by side‐effect profiles and local workflow considerations rather than expectations of different rescue‐medication needs [33].

In our network meta‐analysis, IN ketamine was associated with a significantly higher risk of adverse events than IN fentanyl (RR 1.92, 95% CI 1.58–2.34). High‐concentration IN fentanyl, IV fentanyl, and IV morphine showed no significant differences relative to standard‐dose IN fentanyl, while mild adverse events were more common with oral hydrocodone/acetaminophen. These findings mirror the PRIME trial [8], where IN ketamine produced a substantially higher adverse‐event rate than IN fentanyl (RR = 2.5), with symptoms largely minor and transient. Similarly, a previous systematic review concluded that IN ketamine provides analgesia comparable to IN fentanyl but increases rates of sedation and nonserious side effects [11]. Mechanistically, ketamine’s noncompetitive NMDA receptor antagonism, along with additional activity at opioid, monoaminergic, and muscarinic receptors, contributes to dissociation, dizziness, dysphoria, nausea, and autonomic effects [34]. IN administration can also produce variable absorption, and rapid mucosal uptake may briefly elevate CNS levels, heightening perceptual side effects [35]. In contrast, fentanyl’s predominantly μ‐opioid activity yields more predictable sedation with fewer neuropsychological symptoms [36]. Because ketamine‐related adverse events in pediatric RCTs are usually mild and self‐limiting, ketamine remains a viable option, though its selection should be deliberate and weighed against its higher side‐effect burden.

While adverse events are important to consider, it is worth noting that mild sedation, often categorized as an adverse effect, may paradoxically facilitate subsequent care in selected patients. For children with significant needle anxiety or needle phobia, the mild sedative effects of IN ketamine or fentanyl may improve cooperation and ease the establishment of intravenous access when additional analgesia or interventions are required. This dual benefit—analgesia plus anxiolysis—may be particularly valuable in the pediatric emergency setting, where procedural distress is a major barrier to timely care [10]. However, this potential benefit must be balanced against the risks of over‐sedation and the need for appropriate monitoring.

For acceptability, our analysis showed no significant differences between IN esketamine or nitrous oxide and IN dexmedetomidine in either parent‐reported or provider‐reported outcomes. These findings align with earlier evidence demonstrating the strong influence of route on acceptability. In the multicenter trial by Kendall et al. [19], IN diamorphine was far more acceptable to both providers (98% vs 32%) and parents (97% vs 72%) compared with IM morphine, illustrating that needle avoidance exerts a large, route‐dependent effect. Recent pediatric trials from Nikula et al. [18] also reported no differences in acceptability between IN dexmedetomidine, IN esketamine, and nitrous oxide, reinforcing our results. Contemporary pediatric emergency medicine reviews similarly note that families prefer noninvasive routes and that visible side effects, particularly ketamine‐associated dysphoria or prolonged somnolence, can reduce satisfaction [37]. Thus, when acceptability is a priority, IN delivery is advantageous, and agents with fewer psychoperceptual effects (for example, fentanyl and dexmedetomidine) or rapid‐recovery agents like nitrous oxide may offer the best immediate patient and caregiver experience.

LOS data were available from two trials, each evaluating different agents and clinical contexts. In the randomized pilot trial by Maki et al. [21], children receiving IN lidocaine for acute migraine had an ED stay comparable to those receiving placebo (mean LOS 175.3 ± 84.1 vs. 196.8 ± 118.6 min), suggesting that IN lidocaine does not prolong throughput. However, lidocaine’s lack of significant effect on LOS is not unexpected given its distinct pharmacologic profile. Unlike opioids or ketamine, lidocaine exerts predominantly local anesthetic effects with minimal systemic sedation or neuropsychiatric effects, and its primary benefit is in migraine‐specific pain modulation rather than broad procedural sedation. In contrast, the feasibility RCT by Reynolds et al. [22] comparing IN ketamine with IN fentanyl for extremity injuries reported median times from ED arrival to discharge that were clinically similar (approximately 304 vs 327 min), indicating no operational disadvantage with either IN agent. Collectively, these findings suggest that IN analgesia is operationally efficient and improves comfort without meaningfully impacting overall ED throughput, though the mechanisms underlying LOS neutrality may differ by agent.

This meta‐analysis possesses several notable strengths that enhance the validity and clinical relevance of its findings. By synthesizing data from 15 RCTs encompassing 1388 pediatric patients, the study provides one of the most comprehensive comparative evaluations of IN and intravenous analgesic and sedative agents in children to date. The use of a network meta‐analytic framework allows for integration of both direct and indirect comparisons, thereby enabling relative ranking of treatments even when head‐to‐head data are sparse. An additional strength of this review is the broad range of clinical indications for which IN analgesia demonstrated effectiveness, including extremity fractures, burns, lacerations, migraine headaches, sickle cell vaso‐occlusive crisis, painful infectious mouth lesions, and procedural sedation. This heterogeneity in clinical contexts strengthens the generalizability of our findings and underscores the versatility of the IN route across diverse pediatric emergency presentations.

Despite these strengths, several limitations warrant consideration. The included trials varied in dosing regimens, timing of pain assessments, choice of pain scoring systems (e.g., VAS, FPS‐R, and NRS), and the clinical thresholds for administering rescue analgesia. Specifically, the doses used for different agents were not always pharmacologically equivalent, and bioequivalence assumptions between IN and intravenous routes were not consistently validated, which may affect comparative effectiveness estimates. This introduces clinical heterogeneity that may not be fully captured by statistical models. Some studies had small sample sizes or pilot design structures, limiting power to detect modest differences, particularly in rare or episodic secondary outcomes such as adverse events or rescue medication use. Adverse event reporting was inconsistent across trials, with some capturing only predefined or clinically significant events, while others reported all mild symptoms; this discrepancy may have inflated pooled RRs for agents like ketamine. Blinding challenges, especially in comparisons of IN versus intravenous drugs, could have introduced performance and detection biases. Additionally, few trials evaluated recently emerging agents such as IN dexmedetomidine or esketamine, restricting the robustness of comparisons involving these drugs. Finally, several estimates, particularly for acceptability and LOS, were informed by only two to three studies, which necessitates cautious interpretation of findings.

The findings of this meta‐analysis have important implications for pediatric emergency and acute pain management. The consistent equivalence in analgesic efficacy between IN fentanyl, IN ketamine, intravenous opioids, and other IN agents across all measured timepoints supports the clinical shift toward IN‐first analgesia protocols [38]. The noninvasive nature of IN delivery, coupled with its rapid absorption through the richly vascularized nasal mucosa, allows children to receive timely analgesia without the delays, discomfort, or procedural burden associated with intravenous cannulation [11]. IN fentanyl appears to offer the most favorable balance of efficacy and tolerability, making it a strong first‐line option when opioid therapy is appropriate [26]. IN ketamine provides an opioid‐sparing alternative that maintains analgesic equivalence, though its higher rate of perceptual or neuropsychiatric adverse effects necessitates careful patient selection and anticipatory counseling for caregivers [39]. The high levels of acceptability among parents and providers across all IN agents, combined with neutral or modestly improved impacts on ED LOS, reinforce the practicality of these regimens in fast‐paced clinical environments. Taken together, these findings support broader adoption of evidence‐based IN analgesia pathways that emphasize rapid relief, patient comfort, and operational efficiency.

Further research is essential to refine and extend these findings. Large, multicenter randomized trials are needed to better delineate optimal dosing strategies, particularly for agents with variable IN bioavailability such as ketamine. Comparative trials involving newer pharmacologic options, including IN dexmedetomidine, esketamine, and sufentanil, would enable more definitive ranking of modern IN agents beyond the traditional fentanyl–ketamine framework. Pharmacokinetic and pharmacodynamic modeling studies would help clarify age‐specific, weight‐adjusted, and mucosal absorption–dependent differences in drug exposure, potentially guiding individualized therapy. Additional attention should be directed toward combination IN regimens that may mitigate adverse effects while preserving or enhancing analgesic potency. Future trials should also incorporate validated metrics for caregiver‐reported and child‐centered outcomes, including anxiety, distress reduction, and return to function, in addition to traditional pain scores. Finally, implementation science and cost‐effectiveness studies will be critical to understanding how IN‐first protocols perform in varied EDs and urgent care settings and whether they translate to meaningful improvements in workflow, patient satisfaction, and resource utilization in routine practice.

## 5. Conclusion

In this systematic review and network meta‐analysis of RCTs involving 1388 pediatric patients, IN analgesics demonstrated noninferior analgesic efficacy compared with intravenous and other IN agents across all evaluated timepoints in children presenting with acute pain to EDs. No agent showed statistically superior pain reduction compared with IN fentanyl, and requirements for rescue analgesia were similarly equivalent across treatments. These findings indicate that IN delivery provides reliable and timely analgesia that is clinically comparable to parenteral routes in the acute pediatric emergency setting.

Notably, differences emerged in safety profiles. IN ketamine was associated with a higher incidence of adverse events compared with standard‐dose IN fentanyl, although these events were predominantly mild and transient. Acceptability among patients, caregivers, and providers was high across IN agents, and available data suggest no meaningful impact on ED LOS. Together, these findings support IN fentanyl as a well‐tolerated and operationally efficient first‐line option for the management of moderate to severe acute pain in children, with IN ketamine serving as a viable opioid‐sparing alternative when used judiciously.

Overall, the results reinforce the role of IN‐first analgesia‐focused strategies in pediatric emergency care, offering a noninvasive, effective, and patient‐centered approach that minimizes procedural distress without compromising analgesic efficacy. Future multicenter trials with standardized dosing, consistent adverse‐event reporting, and robust evaluation of operational and patient‐centered outcomes are needed to further optimize IN analgesia protocols and guide evidence‐based implementation across diverse emergency care settings.

Notably, differences emerged in safety profiles. IN ketamine was associated with a higher incidence of adverse events compared with standard‐dose IN fentanyl, although these events were predominantly mild and transient. Acceptability among patients, caregivers, and providers was high across IN agents, and available data suggest no meaningful impact on ED LOS. Together, these findings support IN fentanyl as a well‐tolerated and operationally efficient first‐line option for the management of moderate to severe acute pain in children, with IN ketamine serving as a viable opioid‐sparing alternative when used judiciously.

Overall, the results reinforce the role of IN‐first analgesia strategies in pediatric emergency care, offering a noninvasive, effective, and patient‐centered approach that minimizes procedural distress without compromising analgesic efficacy. Future multicenter trials with standardized dosing, consistent adverse‐event reporting, and robust evaluation of operational and patient‐centered outcomes are needed to further optimize IN analgesia protocols and guide evidence‐based implementation across diverse emergency care settings.

**Nomenclature**
ED: Emergency department
IN: Intranasal
IV: Intravenous
IM: Intramuscular
RCT: Randomized controlled trial
VAS: Visual analog scale
FPS‐R: Faces pain scale–revised
NRS: Numeric rating scale
LOS: Length of stay
RR: Risk ratio
MD: Mean difference
CI: Confidence interval
RoB: Risk of bias
SUCRA: Surface Under the Cumulative Ranking Curve

## Author Contributions

Mohammed Alsabri conceived and designed the study, developed the study protocol, coordinated all stages of manuscript development, supervised the research team, drafted substantial portions of the manuscript, and managed all communications with co‐authors. Yahya A. Mahmoud performed data analysis and contributed to interpretation of the results. Shree Rath led the literature screening, PROSPERO registration, creation of the data extraction sheet, and finalization of included studies and contributed to validation, data extraction, drafting of the discussion, GRADE assessment, and full manuscript review. Eslam Abady contributed to data extraction and quality assessment, managed the submission process, and did all revisions, either major or minor. Mohamed Ashraf Shehab contributed to data extraction and quality assessment. Raseel Sleem drafted the introduction and methods sections and compiled the manuscript files. Luis L. Gamboa and Mohammed Hamzah critically reviewed, edited, and approved the final version of the manuscript. Michael Gillman provided critical revisions to the manuscript, contributed to the interpretation of clinical implications, and approved the final version.

## Funding

The authors received no specific funding for this work.

## Disclosure

All authors read and approved the final manuscript.

## Ethics Statement

This study is a systematic review and meta‐analysis of previously published randomized controlled trials and did not involve direct interaction with human participants or access to identifiable individual‐level data.

## Consent

The authors have nothing to report.

## Conflicts of Interest

The authors declare no conflicts of interest.

## Supporting Information

Additional supporting information can be found online in the Supporting Information section.

## Supporting information


**Supporting Information** Supporting Table S1. Dosing regimens of all included studies, detailing agent, concentration, dose, volume, and bioavailability considerations. Supporting Figure S1. Detailed risk of bias assessment for each trial using the Cochrane ROB‐2 tool, presented as a traffic‐light plot. Supporting Figure S2. Forest plot of the direct comparison between intranasal fentanyl and intranasal ketamine for pain reduction at the 60‐min timepoint. Supporting Figure S3. Forest plot of the direct comparison between intranasal diamorphine and intramuscular morphine for the need for rescue analgesia.

## Data Availability

Data sharing is not applicable to this article as no datasets were generated or analyzed during the current study.
