# Supplementary material for: Use of Intranasal Analgesia for Acute Pain in Pediatric Emergency Departments: A Systematic Review and Meta‐Analysis of Randomized Controlled Trials
Source: Pain Res Manag. 2026 Oct 19;2026:1894053. doi: 10.1155/prm/1894053 (PMC13678576; doi:10.1155/prm/1894053)
Supplement: Supplementary file 1 [file PRM-2026-1894053-s001.docx]

**Table S1: Dosing Regimens of Included Studies**

| Study | Agent | Concentration | Dose | Volume | Bioavailability Consideration |
| --- | --- | --- | --- | --- | --- |
| Frey et al. (2019) [8] | IN Fentanyl | 50 mcg/mL | 1.5-2.0 mcg/kg | 0.03-0.04 mL/kg | ~70-80% bioavailability |
| Frey et al. (2019) [8] | IN Ketamine | 100 mg/mL | 1 mg/kg | 0.01 mL/kg | ~45-50% bioavailability |
| Graudins et al. (2015) [13] | IN Fentanyl | 50 mcg/mL | 1.5 mcg/kg | 0.03 mL/kg | ~70-80% bioavailability |
| Graudins et al. (2015) [13] | IN Ketamine | 100 mg/mL | 1 mg/kg | 0.01 mL/kg | ~45-50% bioavailability |
| Reynolds et al. (2017) [15] | IN Fentanyl | 50 mcg/mL | 2.0 mcg/kg | 0.04 mL/kg | ~70-80% bioavailability |
| Reynolds et al. (2017) [15] | IN Ketamine | 100 mg/mL | 1 mg/kg | 0.01 mL/kg | ~45-50% bioavailability |
| Borland et al. (2007) [17] | IN Fentanyl | 50 mcg/mL | 1.5 mcg/kg | 0.03 mL/kg | ~70-80% bioavailability |
| Borland et al. (2007) [17] | IV Morphine | - | 0.1 mg/kg | - | 100% bioavailability |
| Borland et al. (2011) [18] | IN Fentanyl (Std) | 50 mcg/mL | 1.5 mcg/kg | 0.03 mL/kg | ~70-80% bioavailability |
| Borland et al. (2011) [18] | IN Fentanyl (High) | 150 mcg/mL | 3.0 mcg/kg | 0.02 mL/kg | ~70-80% bioavailability |
| Quinn et al. (2021) [16] | IN Fentanyl | 50 mcg/mL | 1.5 mcg/kg | 0.03 mL/kg | ~70-80% bioavailability |
| Quinn et al. (2021) [16] | IN Ketamine | 100 mg/mL | 1 mg/kg | 0.01 mL/kg | ~45-50% bioavailability |
| Fein et al. (2017) [12] | IN Fentanyl | 50 mcg/mL | 1.5-2.0 mcg/kg | 0.03-0.04 mL/kg | ~70-80% bioavailability |
| Maki et al. (2022) [14] | IN Lidocaine | 40 mg/mL (4%) | 4 mg/kg | 0.1 mL/kg | Local effect |
| Tsze et al. (2022) [20] | IN Ketorolac | 30 mg/mL | 15 mg total | 0.5 mL | ~80% bioavailability |
| Tsze et al. (2022) [20] | IV Ketorolac | - | 0.5 mg/kg (max 15 mg) | - | 100% bioavailability |
| Wilson et al. (1997) [21] | IN Diamorphine | - | 0.1 mg/kg | - | ~50-60% bioavailability |
| Kendall et al. (2001) [22] | IN Diamorphine | - | 0.1 mg/kg | - | ~50-60% bioavailability |
| Nikula et al. (2024) [24] | IN Dexmedetomidine | - | 3 mcg/kg | - | ~80% bioavailability |
| Nikula et al. (2024) [24] | IN Esketamine | - | 1 mg/kg | - | ~50% bioavailability |
| Ruffin et al. (2022) [23] | IN Fentanyl | 50 mcg/mL | 1.5-2.0 mcg/kg | 0.03-0.04 mL/kg | ~70-80% bioavailability |
| Ray et al. (2002) [19] | IN Fentanyl | 50 mcg/mL | 1.5-2.0 mcg/kg | 0.03-0.04 mL/kg | ~70-80% bioavailability |

**Figure S1.** Detailed risk of bias assessment for each trial using ROB-2.

**Figure S2.** Forest plot of intranasal fentanyl versus intranasal ketamine for pain reduction at 60 minutes.

**Figures S3.** Forest plot of intranasal diamorphine versus intramuscular morphine for rescue medications.
